# Variability in estimated gene expression among commonly used RNA-seq pipelines

**DOI:** 10.1038/s41598-020-59516-z

**Published:** 2020-02-17

**Authors:** Sonali Arora, Siobhan S. Pattwell, Eric C. Holland, Hamid Bolouri

**Affiliations:** 1 Division of Human Biology, Fred Hutchinson Cancer Research Center, Seattle, WA 98109 USA; 2 Present Address: Benaroya Research Institute, Seattle, WA 98109 USA

**Keywords:** Transcriptomics, Data processing

## Abstract

RNA-sequencing data is widely used to identify disease biomarkers and therapeutic targets using numerical methods such as clustering, classification, regression, and differential expression analysis. Such approaches rely on the assumption that mRNA abundance estimates from RNA-seq are reliable estimates of true expression levels. Here, using data from five RNA-seq processing pipelines applied to 6,690 human tumor and normal tissues, we show that nearly 88% of protein-coding genes have similar gene expression profiles across all pipelines. However, for >12% of protein-coding genes, current best-in-class RNA-seq processing pipelines differ in their abundance estimates by more than four-fold when applied to exactly *the same samples* and *the same set* of RNA-seq reads. Expression fold changes are similarly affected. Many of the impacted genes are widely studied disease-associated genes. We show that impacted genes exhibit diverse patterns of discordance among pipelines, suggesting that many inter-pipeline differences contribute to overall uncertainty in mRNA abundance estimates. A concerted, community-wide effort will be needed to develop gold-standards for estimating the mRNA abundance of the discordant genes reported here. In the meantime, our list of discordantly evaluated genes provides an important resource for robust marker discovery and target selection.

## Introduction

RNA-sequencing data provide a quantitative view of the transcriptome which can be used to study human diseases. Analyzing RNA-seq data from large scale studies requires efficient and uniform processing of raw sequencing reads from all the samples, while controlling for platform-related biases and noise.

In recent years, a number of ground-breaking projects have processed RNA-seq data from large scale studies such as The Cancer Genome Atlas (TCGA)^1^ (RNA-seq data for 10,340 individuals across 33 cancer types), and the Genotype-Tissue Expression Project (GTEx)^2^ (9,662 samples from 551 healthy individuals and 54 body sites). GTEx RNA-seq data processed using the TOPMed pipeline (https://github.com/broadinstitute/gtex-pipeline) was one of the first such efforts. Later, uniformly processed cancer genomic data (RNA-seq, copy number, mutation calls) from various large-scale cancer studies including TCGA were made available (https://portal.gdc.cancer.gov/) by the NCI Genomic Data Commons (GDC)^3^.

Beyond these efforts, Rahman^4^ *et al*., re-processed raw sequencing reads from all TCGA samples to reduce technical inter-sample variability. They provide a readily accessible resource, which provides both normalized and integer gene counts for downstream analysis.

The Expression Atlas^5^ project at the European Bioinformatics Institute likewise uniformly processed publicly available and controlled RNA-Seq data from Array Express, NCBI’s Gene Expression Omnibus (GEO), European Nucleotide Archive (ENA) and various large scale studies such as GTEx. Uniformly processed data are available via the project website and through the Bioconductor package Expression Atlas.

Collado-Torres^6^ *et al*., developed *recount2*, where >4.4 trillion RNA-seq raw reads from >70,000 human samples deposited in TCGA, GTEx and Short Read Archive (SRA) were processed uniformly using the Rail-RNA pipeline^7^. Raw sequencing reads from all studies were processed identically to maximize cross-sample comparability. The resulting “ready-to-analyze summaries” contain expression data for genes, exons, and exon-exon splice junctions, and can be downloaded via an easy to search web interface (https://jhubiostatistics.shinyapps.io/recount/) or R/Bioconductor package^8^.

Vivian^9^ *et al*., developed Toil, a robust workflow which processed >20,000 RNA-seq samples from four large scale studies (including TCGA and GTEx) in under four days using 32,000 commercial cloud-computing preemptable cores. They demonstrated a 30-fold reduction in cost and time compared to existing pipelines which process similar large-scale datasets. Additionally, their workflow can be shared easily using docker containers, increasing reproducibility and reducing the burden on the researcher to install and configure individual tools.

Wang *et al*.^10^, used RNA quality information to select and process TCGA and GTEx samples not affected by RNA-degradation issues using two different approaches. One approach minimizes differences between normal tissues from TCGA and matching tissues from GTEx. The second approach (used in this paper) uniformly processes both data sets without the inter-project normalization step.

Each of these pipelines, which re-process raw sequencing RNA-seq reads from large scale studies are valuable in their own right and provide widely-used gene expression data for scientific research. However, each of these pipelines use different bioinformatic tools and were also aligned to different assemblies of the human genome using diverse genomic annotations. We compared gene expression values from common samples (4,800 tumor samples from TCGA and 1,890 normal-tissue samples from GTEx) processed by the pipelines to understand how gene expression quantification is impacted by differences in data processing. We show that although the majority of protein coding genes show arguably acceptable concordance between pipelines, for >12% of protein coding genes in the genome, different pipelines produce gene expression estimates that are different by more than four-fold.

## Results

**Figure 1:**
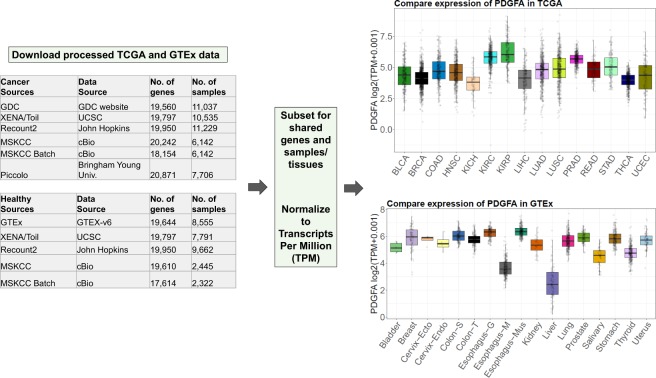
Overview of the data analyzed, showing how data were normalized to allow comparisons among data sets, and example expression data for one gene from GDC and GTEx (v6). Tables show number of genes and samples available from each data source for TCGA and GTEx, Boxplots represent log2(TPM +0.001) values for gene PDGFA for different types of cancer in TCGA and different regions in GTEx. (Abbreviations expanded in Supplementary Table S1c).

To explore the effects of RNA-seq data processing differences on gene expression estimates, we downloaded pan-tissue uniformly-processed RNA-seq abundance values from five different best-in-class processing pipelines for 4,800 tumor samples from TCGA, and calls from four pipelines for 1,890 normal-tissue samples from GTEx. For completeness, we also included an additional dataset for the same samples with batch effect correction between TCGA and GTEx. To ensure fair comparisons, we limited all our analyses to protein coding genes that appear across all data sets (16,109 for TCGA; 16,518 for GTEx). Figure 1 presents an overview of the data sources (details in Supplementary Table S1), their normalization, and example use-case.

It is important to note that all abundance estimates analyzed here are for the same set of RNA-seq reads. Variations between abundance estimates for the same gene and sample are therefore purely the consequence of different data processing software pipelines^11–13^. Differences among the RNA-seq pipelines discussed here include methods/software, software versions, different annotations, different reference assemblies, run-time parameter values, and counting/normalization methods (Supplementary Table S1a)^14^.

Analysis of exon lengths of protein coding genes suggests a wide spread difference in exon lengths from various annotation sources (Supplementary Table S1b). Nonetheless the observed patterns of differential expression cannot be solely due to annotation differences. Datasets with similar gene expression profiles (e.g. Xena/Toil and Piccolo (see Fig. 1a)), are quite distinct in terms of exon lengths (Supplementary Fig. S1b) and datasets with very dissimilar gene expression (e.g. Recount2 and GDC (Fig. 1a)) are highly similar in terms of exon lengths (Supplementary Fig. S1b).

**Figure 2:**
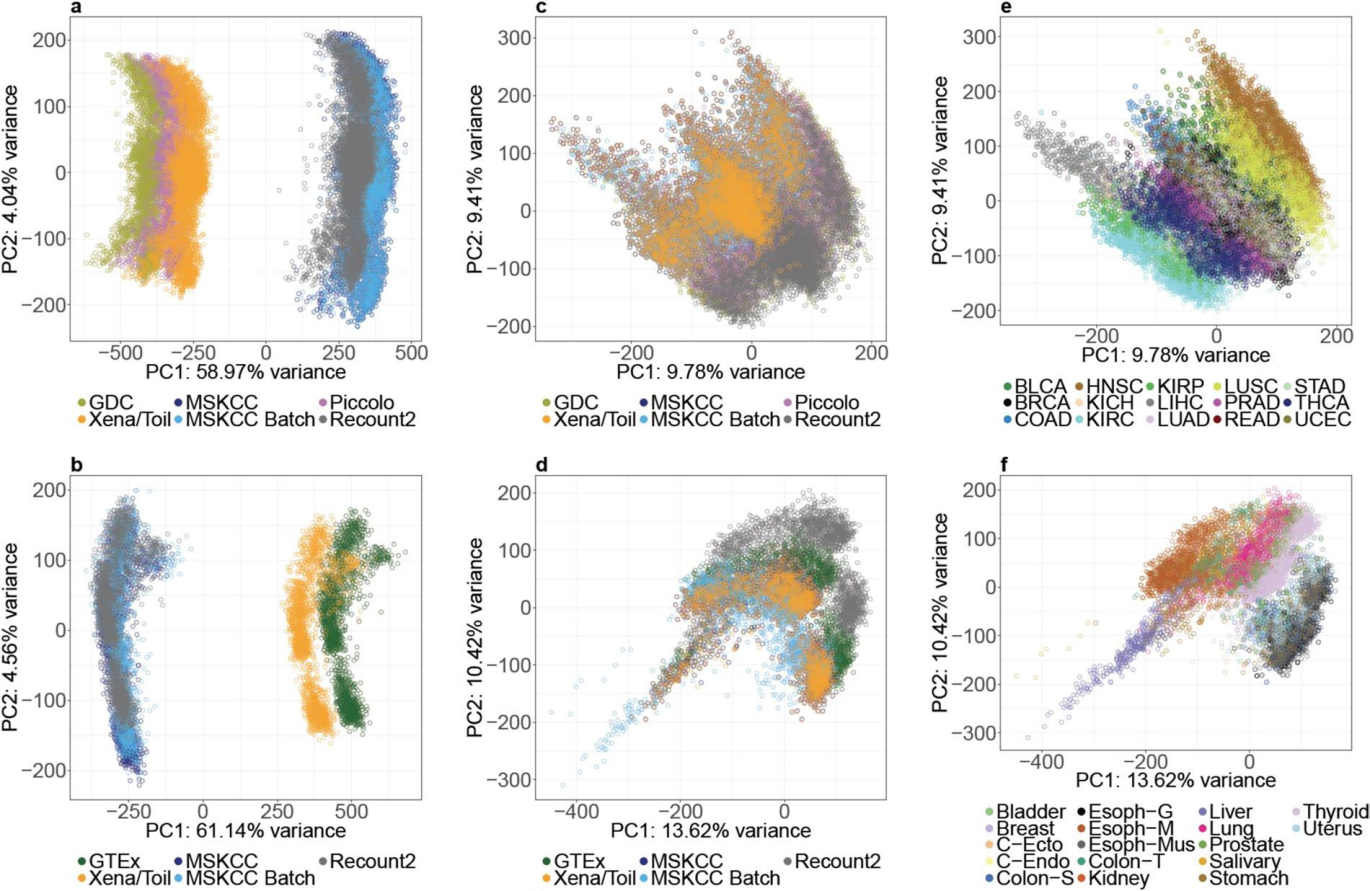
TCGA and GTEx data processed using diverse pipelines exhibit more variation by tissue source than by pipeline. (**a,b**) Principle Component Analysis (PCA) plots of TCGA and GTEx data using RPKM/FPKM values show very large inter-pipeline differences. (**c,d**) After transformation to TPM values, differences among pipelines are dramatically reduced. (**e,f**) Using TPM, variability between tissue types far exceeds inter-pipeline variability for both TCGA and GTEx respectively.

Several of the above data sources are only available in units of fragments per kilobase of transcript per million mapped reads (FPKM)^14^. Because of the way FPKM values are normalized, they can vary between samples^15^, and can be sensitive to differences in reference transcriptome annotations^16^ (Supplementary Table S1). As illustrated in Fig. 2a,b, FPKM-based Principle Component Analysis (PCA) plots of both the TCGA and GTEx data cluster sharply by data source. Indeed, for both TCGA and GTEx data, differences between FPKM based mRNA abundance estimates explain >50% of the total variability in the data (see ‘percent variance explained’ along PC1). To overcome this issue, we converted all data sets to units of transcripts per million (TPM). Because TPM is a fractional abundance measure (per million transcripts), we limited each data set to a common set of 16,738 protein-coding genes before converting FPKM to TPM^14^ (see Online Methods).

After the above unit conversion, the different versions of TCGA and GTEx data appear highly similar at the whole-genome level (Fig. 2c,d and Supplementary Figs. S2, S3), and inter-tissue differences become more pronounced than data-source effects (Fig. 2e,f). Moreover, inter-batch differences do not appear to confound comparisons *within* either TCGA or GTEx data sets, irrespective of data source (Supplementary Figs. S4, S5). As shown in Supplementary Fig. S6 comparisons *between* TCGA and GTEx samples may still be subject to batch effects^10^.

To focus on inter-pipeline differences not related to batch-effects, we analyzed data from the TCGA and GTEx separately and compared only pipelines that do not include batch-effect correction (five sources for TCGA; four sources for GTEx). We searched for genes whose expression in a given sample is >32 TPM according to one pipeline (suggesting high expression), but <8 TPM (i.e. >4-fold lower) in the same sample according to at least one other pipeline. To ensure that such large differences between abundance estimates are not due to a small number of outlier samples, we further required that the expression of a gene should be discordant (i.e. meet the above criteria) in at least 1% of all samples (48 samples for TCGA, 19 samples for GTEx). We refer to genes that meet all the preceding criteria as “discordantly quantified” (DQ) genes.

**Figure 3:**
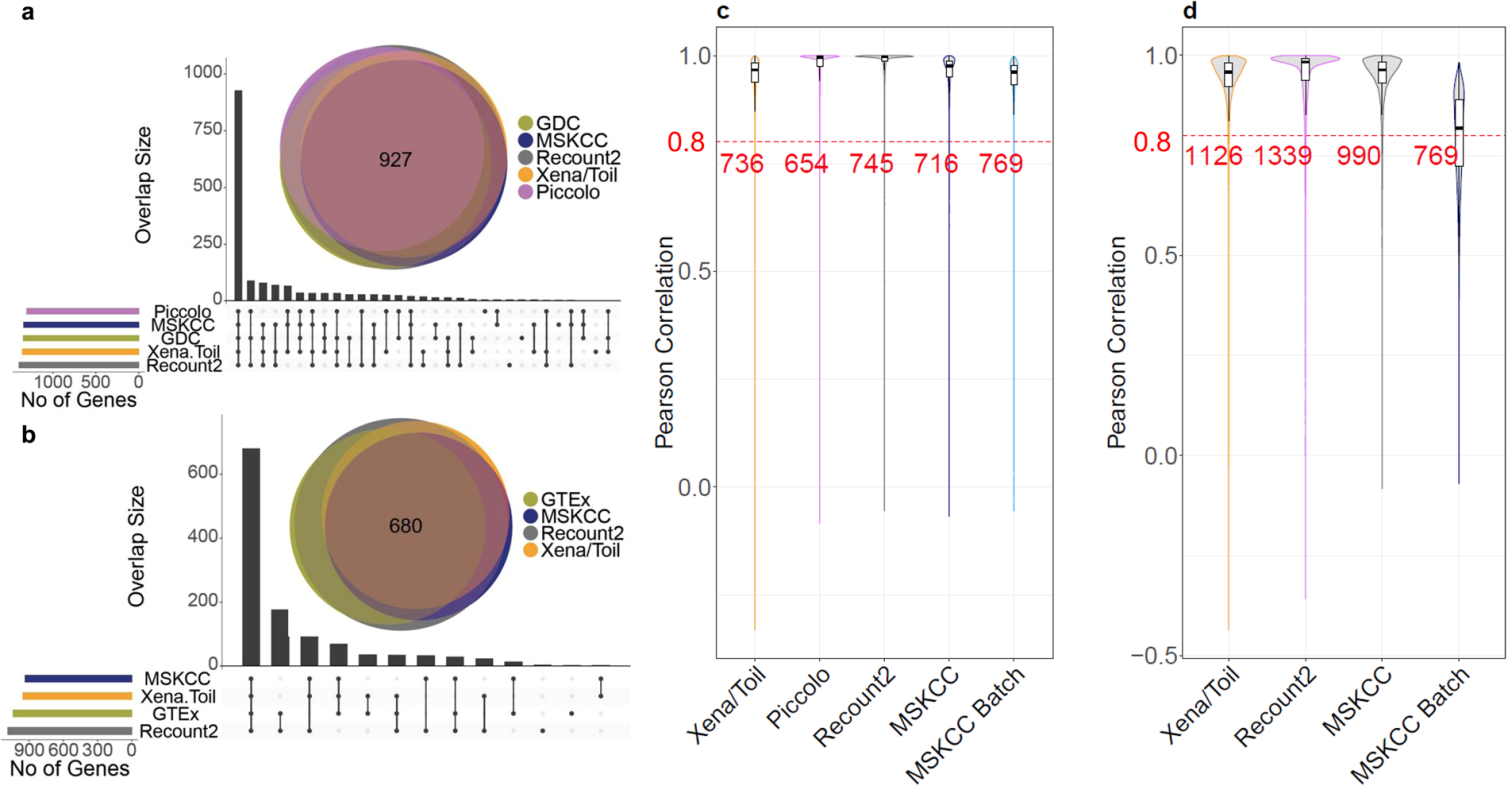
Large-scale inter-pipeline variability in specific genes. (**a,b**) Number of discordant genes per data source for TCGA and GTEx respectively, panels show both UpSet^33^ and Venn diagrams. (**c**) Pairwise Pearson Correlation for gene expression data in TCGA samples comparing five uniformly processed data sources (as labeled) to data from the GDC (GDC-Xena/Toil, GDC-Piccolo, GDC-Recount2, GDC-MSKCC and GDC-MSKCC Batch). **(d)** Pairwise Pearson correlation for gene expression data in GTEx samples from four uniformly processed data sources compared to data from GTEx (GTEx-Xena/Toil, GTEx-Recount2, GTEx-MSKCC, GTEx-MSKCC Batch). Red dashed line represents Pearson correlation = 0.8 and counts represent the number of genes with Pearson correlation > = 0.8.

We found 1,637 DQ genes (~10%) in TCGA data and 1,214 DQ genes (~7%) in GTEx data Importantly, discordant abundance estimates are not due to outlier estimates from a single pipeline. As shown in Fig. 3a,b, most DQ genes are discordant across multiple pipeline-pairs. Across all TCGA and GTEx data sets, of 16,738 genes analyzed, 2,068 genes (12.36%) are DQ (Supplementary Table S2a,b). It should be noted that we imposed very stringent fold-difference criteria in our identification of DQ genes. In particular, three and two-fold expression differences between pipelines affect many more genes: 3551 DQ genes (~22.04%) and 2,621 DQ genes (~15.87%) were found in TCGA and GTEx respectively for three fold expression differences; 9279 DQ genes (~57.6%) and 8061 DQ genes (~48.8%) were found in TCGA and GTEx respectively for two fold expression differences (Supplementary Table S2c,d**)**. In addition to poor inter-pipeline correlations in mRNA abundance (Fig. 3c,d), for approximately half of the TCGA genes with available protein abundance data, mRNA and protein levels show remarkably low levels of correlation (Supplementary Fig. S7, Supplementary Table S6a–c).

**Figure 4:**
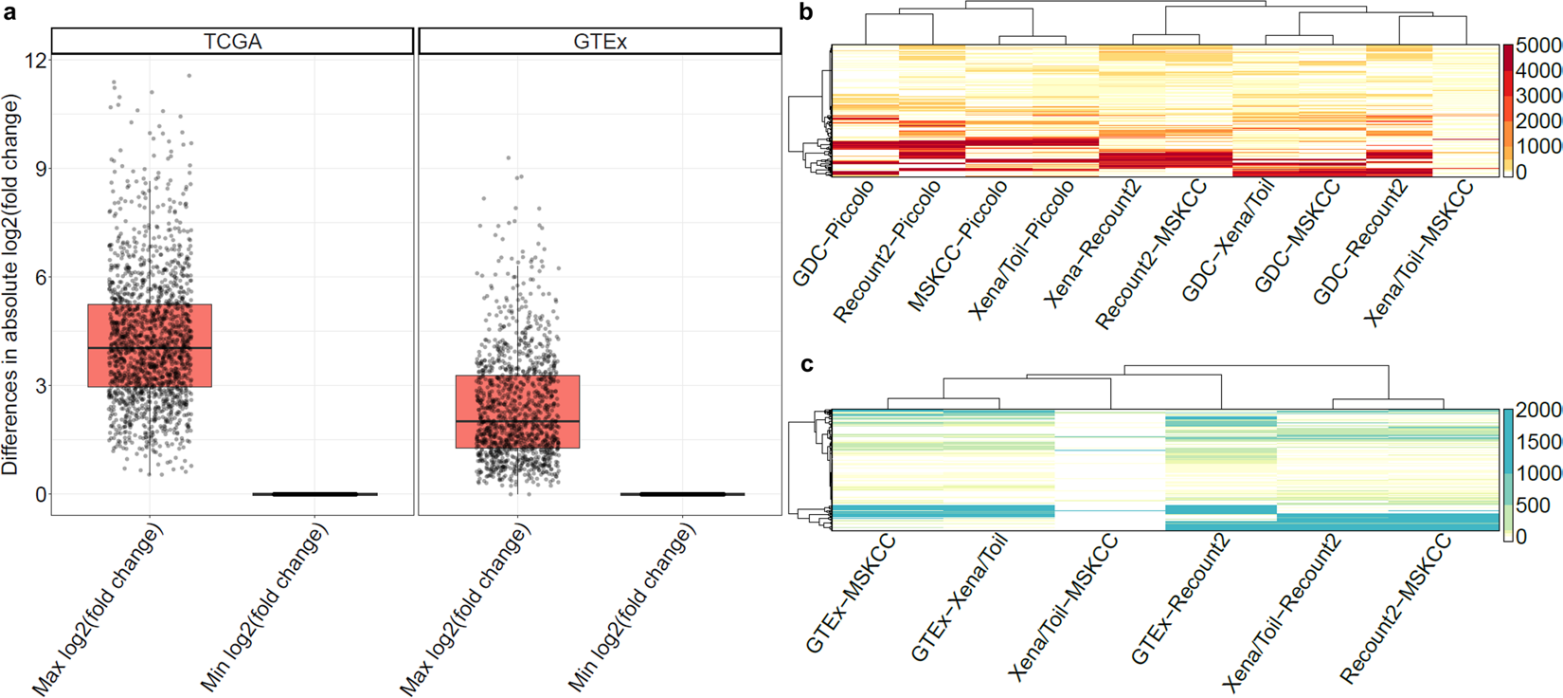
Summaries of fold-change and abundance estimate differences among RNA-seq pipelines. (**a**) Large inter-pipeline differences in fold change estimates for the same sample pairs among discordant genes. Shown are the maximum and minimum fold-difference estimates among pipelines for each discordant gene across all sample pairs. Heatmaps showing the number of discordant samples per gene in TCGA **(b)** and GTEx **(c)** data. Each row represents one discordant gene, each column represents a comparison between two pipelines (as labeled).

The *relative* expression of a given gene between two samples may be expected to be more comparable across pipelines^17^. However, of the above 2,068 DQ genes, 1,958 genes (~95%) have >2-fold inter-pipeline differences in fold-change estimates for the same sample pairs **(**Fig. 4a and Supplementary Table S3**)**. Of note, many DQ genes have divergent expression values in large numbers of samples (>500, Fig. 4b,c and Supplementary Table S4). Importantly, the observed discrepancies are not attributable to a particular subset of processing pipelines or a particular subset of samples. Even the two pipelines with the greatest level of agreement (MSKCC and Xena/TOIL), still include many genes that are DQ in large numbers of samples.

**Figure 5:**
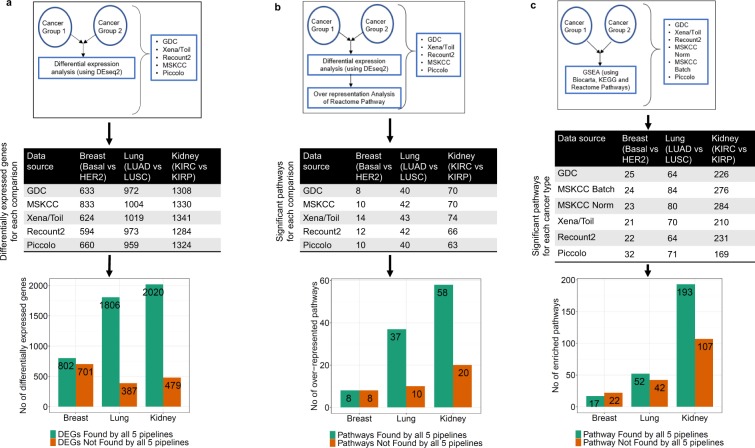
Differential expression and pathway analysis among subtypes of three cancers reveals differences in identified genes and pathways among pipelines. Schematics illustrate (**a**) differential expression analysis (**b**) pathway over-representation analysis and (**c**) Gene Set Enrichment (GSEA) analysis for each cancer comparison. Tables show number of differentially expressed genes/significantly impacted pathways for each comparison across each pipeline. Bar plots show the numbers of differentially expressed genes and pathways found or not-found by all pipelines.

Differences in fold-change estimates affect differential gene expression analysis results^18^. Assessment of differential expression across three cancer subtypes revealed large differences in evaluation outcomes among the five datasets (Fig. 5a). Specifically, between 17% and 47% of differentially expressed genes were not identified as differentially expressed across all five datasets.

Pathway analysis of the samples from the three distinct cancer subtype comparisons using two different approaches also revealed different sets of enriched pathways (Fig. 5b,c, Supplementary Figs. S8–S10, Supplementary Tables S7–S9). Thus, discordantly quantified gene expression values impact common downstream analyses such as differential expression and pathway enrichment, raising further concerns regarding the use of RNA-seq data for biomarker and target discovery.

Our findings are consistent with those of the Sequencing Quality Control Consortium (SEQC), which found that absolute RNA-seq abundance estimates were generally not trustworthy, and that reliable differential expression analysis was only feasible in less than two-thirds of the genome^17^. However, whereas SEQC compared expression data generated using multiple experimental and computational protocols, the discordant expression levels and fold differences reported here are for exactly the same RNA-seq reads and arise entirely from differences in data processing pipelines.

**Figure 6:**
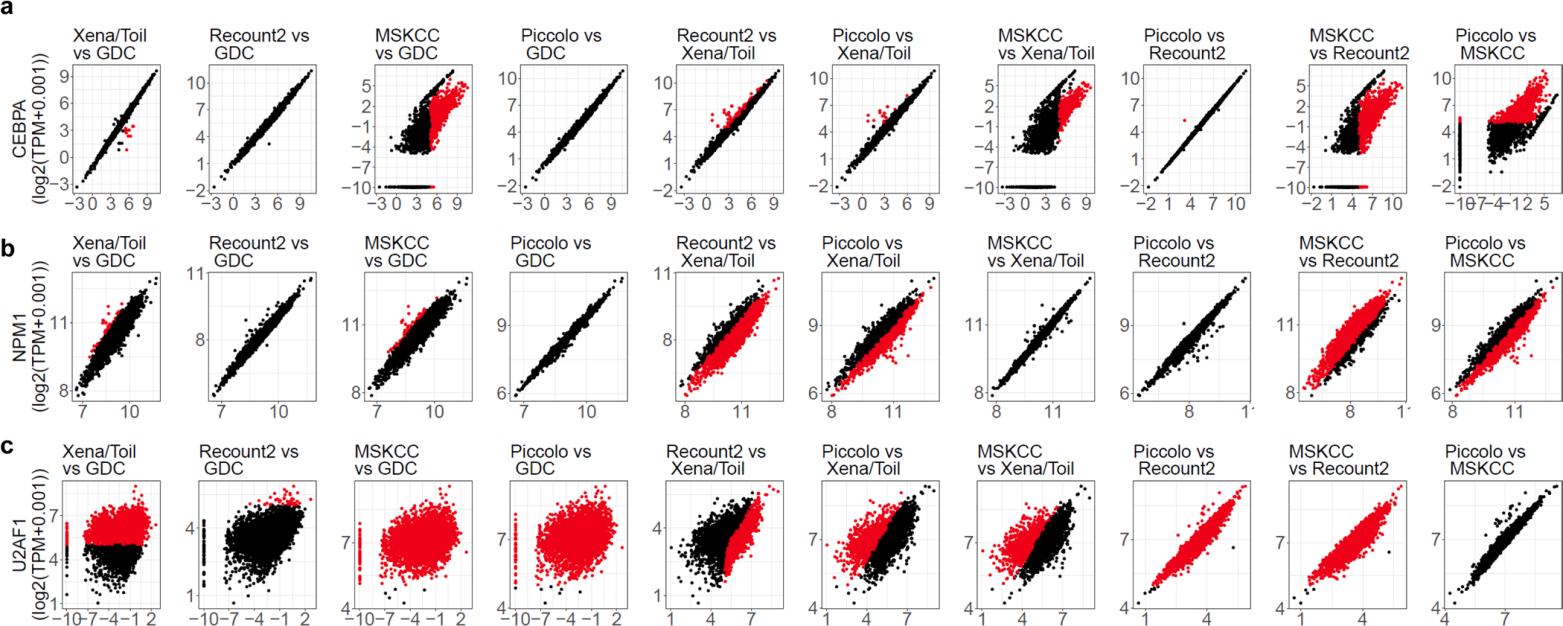
Example pairwise comparisons of expression abundance estimates. Pairwise scatter plots for gene expression data (log2(TPM + 0.001) from (**a**) *CEBPA*, (**b**) *NPM1* and (**c**) *U2AF1* show diverse modes of disagreement among different pipelines. Panel titles specify the pipelines as Y-axis vs. X-axis. Red dots and black dots represent discordant and non-discordant samples respectively between two pipelines.

For most DQ genes, differences between pipelines arise in a variety of ways. As an example, Fig. 6 shows the expression patterns of three DQ genes (the leukemia-associated genes *CEBPA*, and *NPM1*, and the splicing regulator U2 small nuclear RNA auxiliary factor 1 (*U2AF1*), which is frequently mutated in Myelodyplastic Syndrome^19^) in five versions of TCGA data (see our Github repository for additional examples). We note that for *NPM1*, estimates often differ by a simple scaling factor. For *CEBPA* on the other hand, estimate differences appear to be sample-specific. In some pipelines, *U2AF1* is essentially not expressed in a given sample, but highly expressed in the same sample according to another pipeline, demonstrating that abundance estimates for this gene are simply poorly correlated across two pipelines. Depending on which pipeline is used by a researcher to inform subsequent laboratory experiments, caution should be used when designing experiments for DQ genes such as *U2AF1*.

## Discussion

Gene expression data is the starting point for cohort stratification, pathway enrichment analyses, and many other investigations. The five best-in-class RNA-seq pipelines compared here produce consistent results for nearly 88% of protein-coding genes, including many disease-associated genes, such as genes associated with breast cancer (BRCA1, TP53, and ERRB2^20^, Supplementary Fig. S11), prostate cancer (SPOP, IDH1, AKT1^21^, Supplementary Fig. S12), and glioblastoma (SPOP, IDH1, AKT1^22^, Supplementary Fig. S13). By Contrast, abundance estimates for the remaining 12% of protein coding genes (which also include many disease-associated genes), are highly variable across pipelines, suggesting that these genes should be handled with caution in RNA-seq analyses.

The diverse ways in which some abundance estimates vary among data processing pipelines (Fig. 6) suggest that the observed discrepancies do not have a single cause, but rather are driven by multiple factors. Given the large numbers of statistical assumptions, approximations, algorithmic differences, and user-defined run-time parameters inherent in high-throughput data processing, it is not surprising that different pipelines generate different abundance estimates.

One known source of uncertainty in RNA-seq abundance estimates is that more complex genome annotations can increase the numbers of unmapped and multi-mapped reads^16^. Consistent with this observation, 240 of our 2,068 DQ genes (11.61%) are known to be frequently affected by multi-mapping reads (Supplementary Table S2)^23^. Additionally, differing genome annotations have differences in inclusion or exclusion of exons which affects total read counts per gene. Accordingly, 8175 out of 16738 (48.84%) protein coding genes and 1073 out of 2068 DQ (51.88%) genes did not have the same exon count per gene across four of our datasets that use different gencode^24^ annotation versions (Supplementary Table S10).

A previous study comparing the performance of a large number of data processing pipelines to reference transcriptomes for two human cell lines^25^, found low precision and sensitivity in nearly all individual pipelines. Importantly, the authors found that tuning the choices of algorithms and parameters could considerably improve the ability of pipelines to match the refence transcriptome. This finding further highlights the degree to which assumptions underlying RNA-seq data processing pipelines affect their performance.

Our list of 2,068 DQ genes includes 784 disease-associated genes (Supplementary Table S5). Many of these genes (e.g. *CEBPA*, *HIF1A*, and *KRAS*) are widely-studied and play important roles in diverse human tissues and diseases. As such, the finding that their abundance estimates may vary by greater than four-fold depending on the data processing pipeline will be important for biomedical research and translational medicine.

The bioinformatics pipelines compared here represent best-in-class efforts by leading research teams, and utilize well-established, widely used methods. The differences among these pipelines arise from diverse implementation choices including statistical and algorithmic methods, software versions, and run-time parameters. The discordant abundance and fold-change estimates revealed here do not imply any technical errors. Rather they highlight inherent uncertainty in processing noisy and complex data. Nonetheless, the end result is that for the DQ genes reported here (Supplementary Table S2b), we can have little confidence in the abundance estimates produced by any RNA-seq processing pipeline. For critical applications such as biomedical research and clinical practice, a concerted, community-wide effort will be needed to develop gold-standards for estimating the mRNA abundance of these genes.

## Methods

All analyses were performed in R (https://www.r-project.org/) using Bioconductor (https://www.bioconductor.org/) packages. To maximize transparency and reproducibility, we have deposited all scripts, associated data, and a large number of additional plots in a Github repository: https://github.com/sonali-bioc/UncertaintyRNA. R/Bioconductor package TCGAbiolinks^26,27^ was used to download TCGA data from GDC, SummarizedExperiment^28^ was used to store TCGA and GTEx data from each data source, rtracklayer^29^ was used to import GTF files. All plots were made using ggplot2^30^, RcolorBrewer^31^, eulerr^32^, UpSetR^33^ and pheatmap^34^.

### Data sources

RNA Seq gene expression data was downloaded from five and four different sources for TCGA and GTEx respectively (Supplemental Table 1). Each source contained different numbers of genes and samples, thus we included only shared protein coding genes and samples found in every source in our analysis. The batch information for Sequencing Center, Tissue Source Site (TSS) and Plate ID were downloaded from https://gdc.cancer.gov/resources-tcga-users/tcga-code-tables. The nucleic acid isolation batch, genotype and expression batch data for GTEx samples were downloaded from the GTEx website (https://gtexportal.org/home/datasets).

### Conversion of abundance estimates to transcripts per million (TPM)

All data sources except Xena/Toil provided FPKM RNA Seq gene expression data. For consistency, we converted all FPKM gene expression data to TPM data using the formula

$$TPM=\frac{{\rm{FPKM}}}{{\sum }_{allgenes}{\rm{FPKM}}}\,\times \,{10}^{6}$$

as described by Collins *et al*.^35^.

### Principle component analysis (PCA)

Principle Component values were generated using the R function prcomp() using all genes and visualized with R package ggplot2.

### DQ samples

For a gene to be discordant, its expression in at least one data set should be more than 32 TPM (i.e. log2 (TPM+0.001) more than 5) and the log2 fold change should be more than 2 (i.e. >4-fold difference in expression).

### Discordant fold changes

For all sample pairs within each data source, expression fold changes were calculated for discordant genes (as defined above) and compared with fold change differences across other data sources.

### TCGA-GTEx batch effects comparison

Four pipelines provide both TCGA and GTEx data (Supplementary Fig. S5). To visualize potential batch effects between GTEx and TCGA data, we applied Principal Component Analysis to expression data for Stomach/STAD, Liver/LIHC and Thyroid/THCA samples from each pipeline in a manner similar to^2^. In addition to these within pipeline comparisons, we also compared the original versions of the TCGA GDC data and the GTEx (v6) data using the same approach.

### Correlations

Pearson correlation was calculated using rcorr() function from Hmisc R package to compare TPM data from five different sources of TCGA data with the original GDC data for each gene. For protein-mRNA correlations, PANCAN12 protein abundance data was downloaded from https://xenabrowser.net/datapages/?dataset=TCGA.PANCAN12.sampleMap/RPPA_RBN&host=https://tcga.xenahubs.net. We calculated the Spearman correlation between protein levels and gene expression using rcorr() for only those genes for which we had both protein and gene expression data.

### Differential expression and pathway enrichment analysis

For each cancer type, raw counts from each pipeline for the same samples were used for differential expression analysis using DESeq2^36^. A statistical cutoff of fold change >2 and p-adjusted value <0.05 was used to find differentially expressed genes (DEGs). Pathway enrichment analysis using DEGs was done using clusterProfiler^37^ (v 3.4.4). Enrichment results were visualized using dot plots made with ggplot2^30^.

### Gene set enrichment analysis (GSEA)

log2(TPM+0.001) counts from each pipeline were used to conduct a GSEA^38^ analysis using Java GSEA software (v4.0.2, http://software.broadinstitute.org/gsea/downloads.jsp), for all three cancer types.

## Supplementary information

Supplementray information

Supplementary Table S1.

Supplementary Table S2.

Supplementary Table S3.

Supplementary Table S4.

Supplementary Table S5.

Supplementary Table S6.

Supplementary Table S7.

Supplementary Table S8.

Supplementary Table S9.

Supplementary Table S10.

## Data Availability

All data and scripts used in this manuscript are freely available via: https://github.com/sonali-bioc/UncertaintyRNA.
