# Supplementary material for: Variability in estimated gene expression among commonly used RNA-seq pipelines
Source: Sci Rep. 2020 Feb 17;10:2734. doi: 10.1038/s41598-020-59516-z (PMC7026138; doi:10.1038/s41598-020-59516-z)
Supplement: Supplementary file 1 — Supplementray information [file 41598_2020_59516_MOESM1_ESM.pdf]

Variability in estimated gene expression among commonly used RNA-Seq pipelines

Sonali Arora[1], Siobhan S. Pattwell[1], Eric C. Holland[1]*, Hamid Bolouri[1]*#
[1] Division of Human Biology, Fred Hutchinson Cancer Research Center, Seattle, WA 98109, USA

* Correspondence: ehholland@fredhutch.org (E. C. Holland), hbolouri@fredhutch.org (H. Bolouri)

Supplementary Figures

Supplementary Figure S1. (a) Table showing various annotation sources and reference genome used by 5 different pipelines. (b) Principle Component Analysis (PCA) plots of Exon lengths for various annotation.

Supplementary Figure S2. Principle Component Analysis (PCA) plots of TCGA data from (a) GDC and Xena/Toil, (b) GDC and Piccolo Lab, (c) GDC and Recount2, (d) GDC and normalized data from MSKCC (MSKCC) and (e) GDC and batch corrected data, after normalization from MSKCC (MSKCC Batch)

Supplementary Figure S3. Principle Component Analysis (PCA) plots of GTEx data from (a) GTEx and Xena/Toil, (b) GTEx and Recount2, (c) GTEx and normalized data from MSKCC (MSKCC) and (d) GTEx and batch corrected data, after normalization from MSKCC (MSKCC Batch)

Supplementary Figure S4. Principle Component Analysis (PCA) plots of TCGA data from (a) GDC, (b) Xena/Toil, (c) Piccolo Lab (d) Recount2, (e) normalized data from MSKCC (MSKCC) and (f) batch corrected data, after normalization from MSKCC (MSKCC Batch) showing batch effects are not present. Colors in each PCA plot depict batches by “Plate Id”.

Supplementary Figure S5. Uniformly processed GTEx RNA-seq data do not show batch effects. Two batch variables (nucleic acid isolation batch and genotype batch) are available for GTEx data. Principle Component Analysis (PCA) plots of GTEx data from all 5 sources of GTEx data is colored by three nucleic acid batches in red (a) BP-22611, (b) BP-23051, (c) BP-22873 and three genotype batches in purple (d) LCSET-3098 (e) LCSET-3152 and (f) LCSET-3205.

Supplementary Figure S6. Illustration of potential batch effects between TCGA and GTEx. Gene expression data for thyroid, liver and stomach were compared with gene expression data for their respective cancer types (THCA, LIHC and STAD) from TCGA. (a) GDC, (b) Xena/Toil, (c) Recount2, (d) normalized data from MSKCC (MSKCC) and (e) batch corrected data, after normalization from MSKCC (MSKCC Batch) showing reduced differences between TCGA and GTEx.

Supplementary Figure S7. Spearman correlation for gene expression data from TCGA and available protein abundance data.

Supplementary Figure S8. Upset plots showing differentially expressed genes and over-represented Reactome pathways in (a, b) Basal-like subtype compared to HER2-enriched(HER2E) subtype for TCGA-BRCA (c, d) Lung adenocarcinoma(TCGA-LUAD) compared to Lung squamous cell carcinoma (TCGA-LUSC) and (e, f) Kidney renal clear cell carcinoma (TCGA-KIRC) compared to Kidney renal papillary cell carcinoma(TCGA-KIRP) using same samples across all pipelines. Differentially expressed genes were obtained from DESeq2 (FDR < 0.05 & fold change >1).

Supplementary Figure S9. Dot plots showing pathways over-represented in (a) Basal like subtype compared to HER2-enriched subtype of TCGA-BRCA samples , in (b) Lung adenocarcinoma(TCGA-LUAD) compared to Lung squamous cell carcinoma (TCGA-LUSC), and in (c) Kidney renal clear cell carcinoma (TCGA-KIRC) compared to Kidney renal papillary cell carcinoma (TCGA-KIRP) across all pipelines .(x-axis represents each pipeline, y-axis represents pathways, presence/absence of circle represents if the pathway was over-represented in respective pathway, size of circle represents geneRatio whereas color of circle represents the adjusted p-value, “GeneRatio” is the number of genes within the list which are annotated to the gene set divided by the size of list of genes of interest.)

Supplementary Figure S10. Upset plots showing enriched pathways in (a)Basal-like subtype compared to HER2-enriched(HER2E) subtype for TCGA-BRCA (b) Lung adenocarcinoma(TCGA-LUAD) compared to Lung squamous cell carcinoma (TCGA-LUSC) and (c) Kidney renal clear cell carcinoma (TCGA-KIRC) compared to Kidney renal papillary cell carcinoma(TCGA-KIRP) obtained from GSEA analysis of log2(TPM) counts using same samples across all pipelines.

Supplementary Figure S11. (a) BRCA1, (b) TP53 and (c) ERBB2 (genes associated with breast cancer) show agreement among different pipelines. Panel titles specify the pipelines as Y-axis vs. X-axis. Red dots and black dots represent discordant and non-discordant samples respectively between two pipelines.

Supplementary Figure S12. (a) EIF4EBP1, (b) NFKB2 and (c) AKT1 (genes associated with prostate cancer) show agreement among different pipelines. Panel titles specify the pipelines as Y-axis vs. X-axis. Red dots and black dots represent discordant and non-discordant samples respectively between two pipelines.

Supplementary Figure S13. (a) RB1, (b) PIK3CA and (c) EGFR (genes associated with glioblastoma) show agreement among different pipelines. Panel titles specify the pipelines as Y-axis vs. X-axis. Red dots and black dots represent discordant and non-discordant samples respectively between two pipelines.

Supplementary Tables

Supplementary Table S1. (a) Overview of data sources for TCGA and GTEx RNA-Seq data. (b) Exon lengths of protein coding genes from various annotation source used by 5 different pipelines.(c) Approx. 120 models created by varying ref genome, annotation source, alignment software ang gene quantification software to study affects of varied data processing across pipelines.(d) Abbreviations used for TCGA cancer types and GTEx region text and figures.

Supplementary Table S2. (a) Overview of number and percentage of DQ genes at different thresholds. Discordantly quantified (DQ) genes across multiple pipeline-pairs showing (b) four-fold difference (c) three-fold difference (d) two-fold difference for TCGA and GTEx pipelines.

Supplementary Table S3. Large inter-pipeline differences in fold change estimates for the same sample pairs among discordant genes in (a) TCGA and (b) GTEx pipelines.

Supplementary Table S4. Discordant samples which have divergent expression values for DQ genes in (a) TCGA and (b) GTEx pipelines.

Supplementary Table S5. List of Disease-associated DQ genes.

Supplementary Table S6. Pairwise Pearson correlation for gene expression data in (a)TCGA and (b) GTEx samples from four uniformly processed pipelines compared to data from GDC and GTEx respectively. (c) Spearman correlation for gene expression data from TCGA and available protein abundance data.

Supplementary Table S7. Differentially expressed genes in (a) Basal-like subtype compared to HER2-enriched(HER2E) subtype for TCGA-BRCA (b) Lung adenocarcinoma(TCGA-LUAD) compared to Lung squamous cell carcinoma (TCGA-LUSC) and (c) Kidney renal clear cell carcinoma (TCGA-KIRC) compared to Kidney renal papillary cell carcinoma(TCGA-KIRP) across all 5 pipelines.

Supplementary Table S8. Over-represented analysis of Reactome Pathways in (a) Basal-like subtype compared to HER2-enriched(HER2E) subtype for TCGA-BRCA (b) Lung adenocarcinoma(TCGA-LUAD) compared to Lung squamous cell carcinoma (TCGA-LUSC) and (c) Kidney renal clear cell carcinoma (TCGA-KIRC) compared to Kidney renal papillary cell carcinoma(TCGA-KIRP) across all 5 pipelines.

Supplementary Table S9. GSEA analysis of Biocarta, KEGG and Reactome Pathways in (a) Basal-like subtype compared to HER2-enriched(HER2E) subtype for TCGA-BRCA (b) Lung adenocarcinoma(TCGA-LUAD) compared to Lung squamous cell carcinoma (TCGA-LUSC) and (c) Kidney renal clear cell carcinoma (TCGA-KIRC) compared to Kidney renal papillary cell carcinoma(TCGA-KIRP) across all 5 pipelines.

Supplementary Table S10: Number of exons for each protein coding gene from various Gencode annotation sources used by 4 different pipelines.

Supplementary Figure - S1

a

Data Source	Annotation Source	Reference Genome
GDC	Gencode (v 22)	hg38
MSKCC	Gencode (v 22)	hg19
Piccolo	Illumina's GTF	hg19
Recount2	Gencode (v 25)	hg38
Xena/Toil	Gencode (v 23)	hg38

b

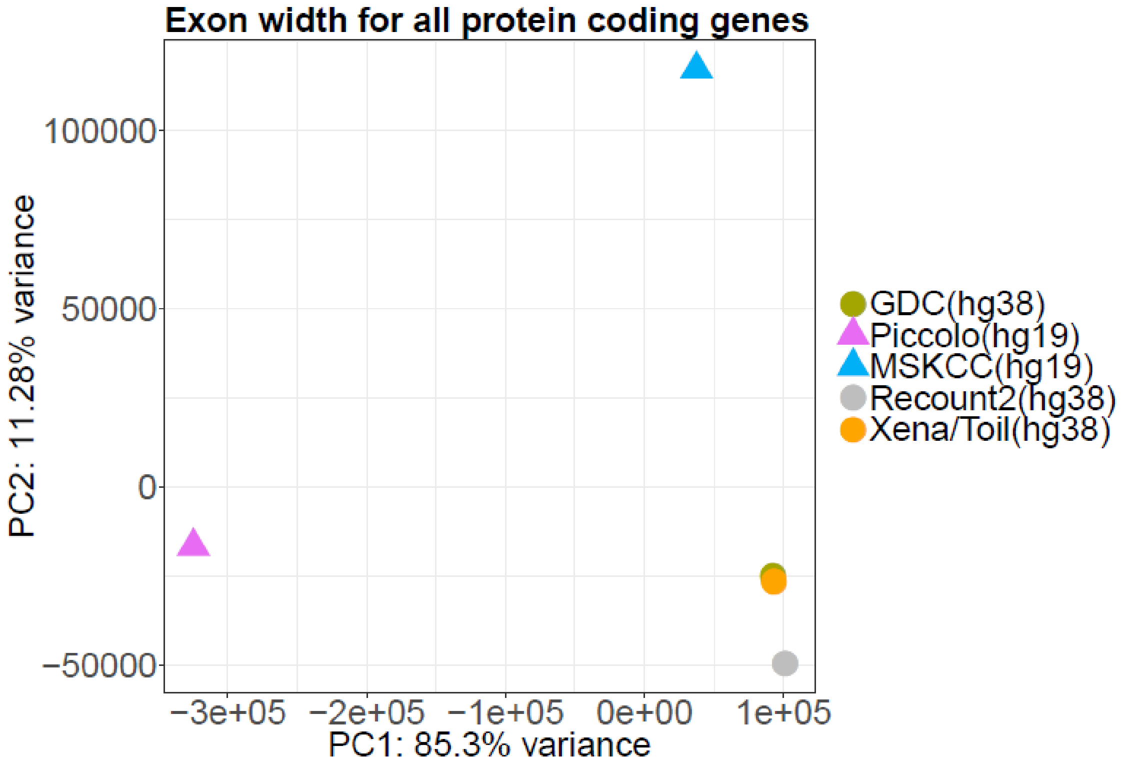

Supplementary Figure S1. (a) Table showing various annotation sources and reference genome used by 5 different pipelines. **(b)** Principle Component Analysis (PCA) plots of Exon lengths for various annotation.

Supplementary Figure - S2

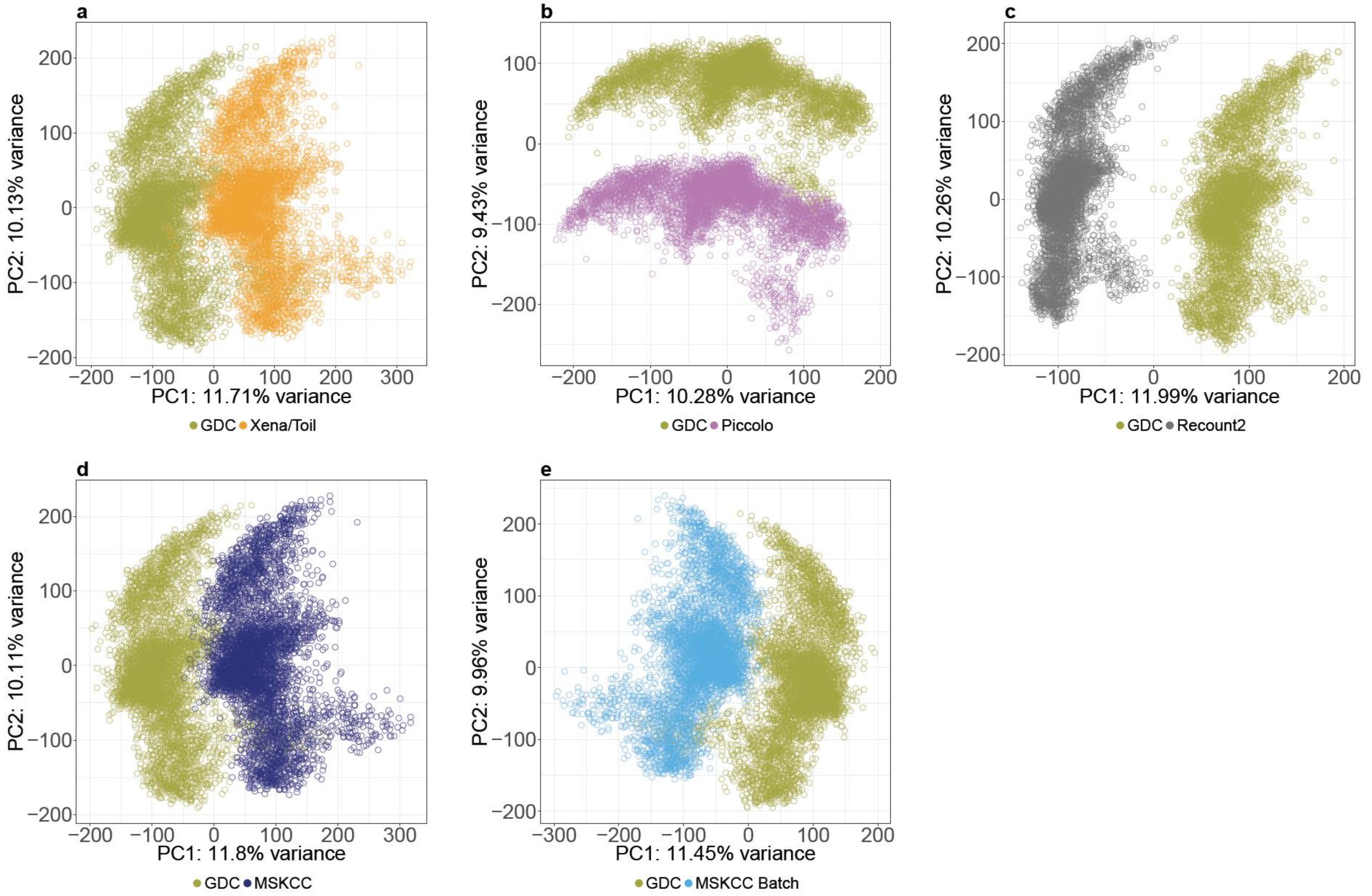

Supplementary Figure S2. Principle Component Analysis (PCA) plots of TCGA data from **(a)** GDC and Xena/Toil, **(b)** GDC and Piccolo Lab, **(c)** GDC and Recount2, **(d)** GDC and normalized data from MSKCC (MSKCC) and **(e)** GDC and batch corrected data, after normalization from MSKCC (MSKCC Batch)

Supplementary Figure - S3

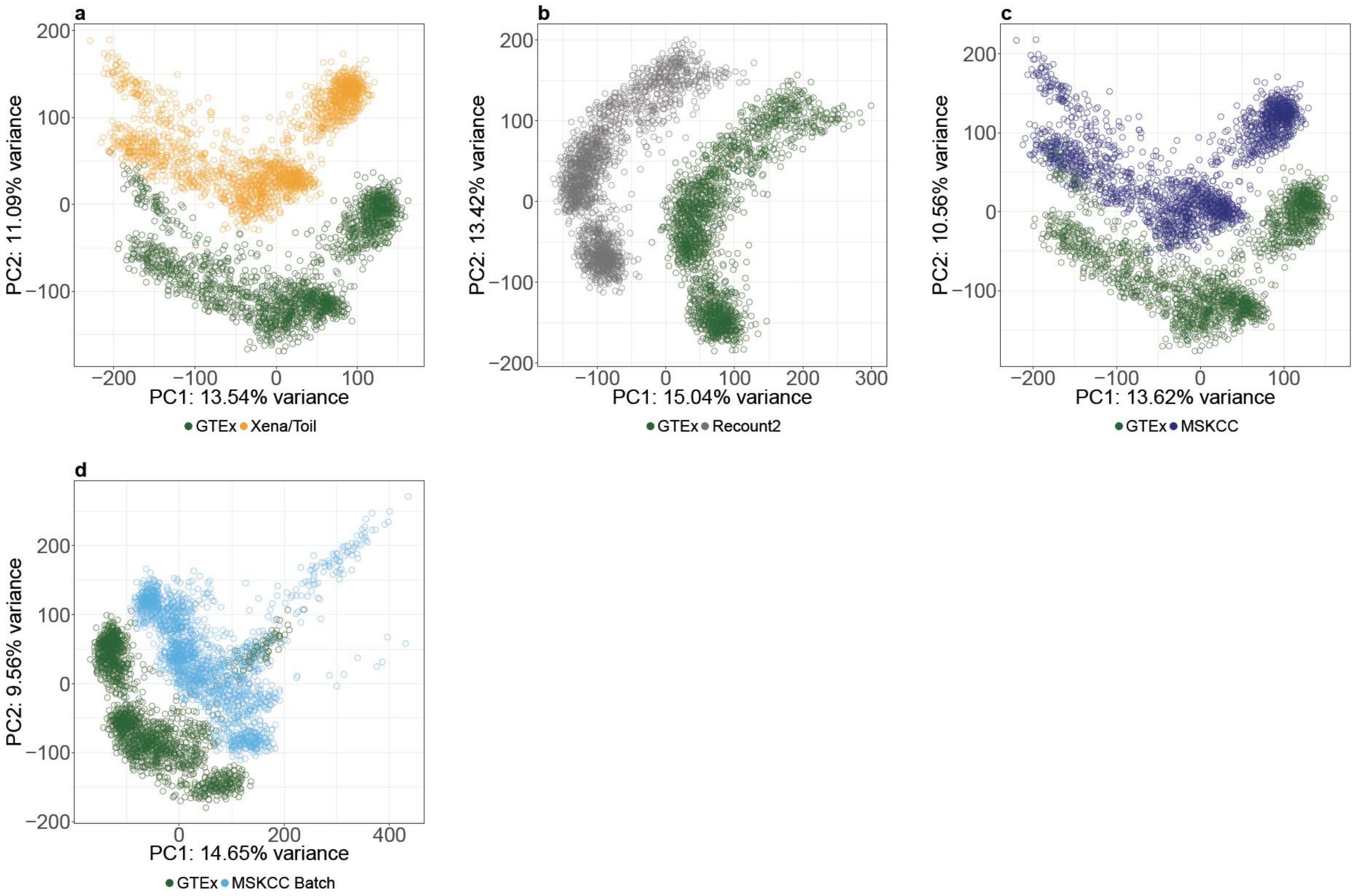

Supplementary Figure S3. Principle Component Analysis (PCA) plots of GTEx data from **(a)** GTEx and Xena/Toil, **(b)** GTEx and Recount2, **(c)** GTEx and normalized data from MSKCC (MSKCC) and **(d)** GTEx and batch corrected data, after normalization from MSKCC (MSKCC Batch)

Supplementary Figure - S4

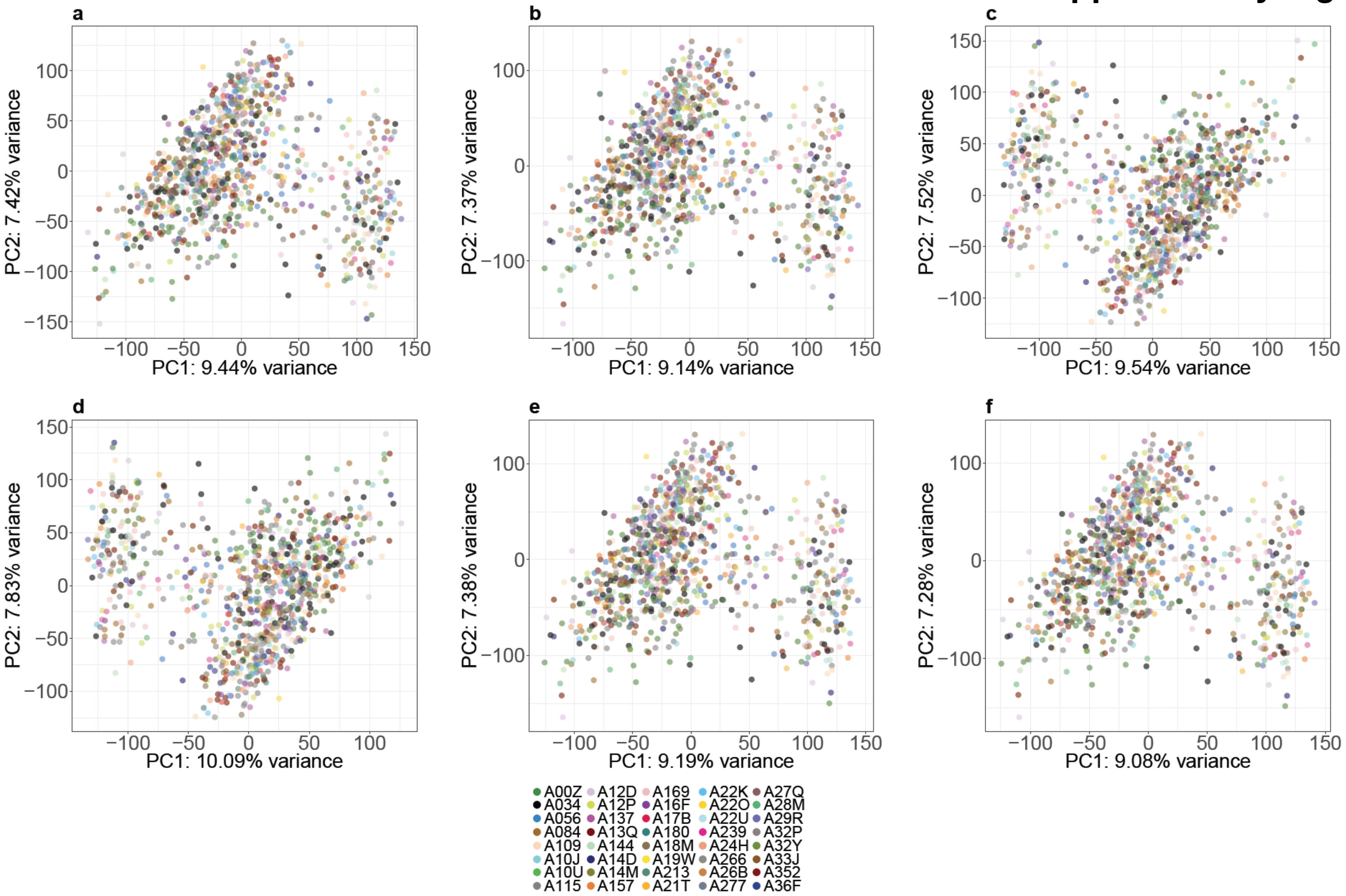

Supplementary Figure S4. Principle Component Analysis (PCA) plots of TCGA data from **(a)** GDC, **(b)** Xena/Toil, **(c)** Piccolo Lab **(d)** Recount2, **(e)** normalized data from MSKCC (MSKCC) and **(f)** batch corrected data, after normalization from MSKCC (MSKCC Batch) showing batch effects are not present. Colors in each PCA plot depict batches by “Plate Id”.

Supplementary Figure - S5

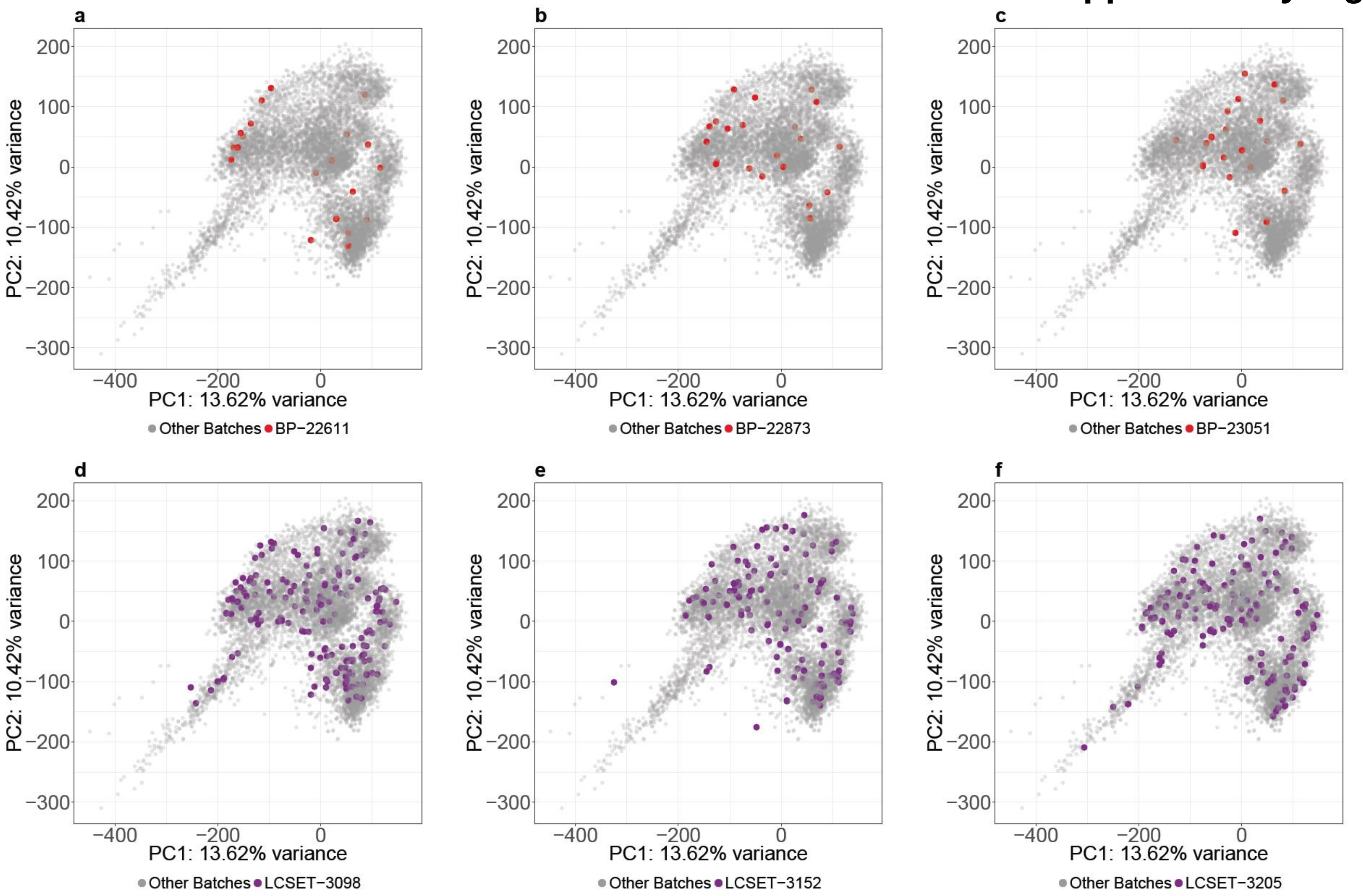

Supplementary Figure S5. Uniformly processed GTEx RNA-seq data do not show batch effects. Two batch variables (nucleic acid isolation batch and genotype batch) are available for GTEx data. Principle Component Analysis (PCA) plots of GTEx data from all 5 sources of GTEx data is colored by three nucleic acid batches in red **(a)** BP-22611, **(b)** BP-23051, **(c)** BP-22873 and three genotype batches in purple **(d)** LCSET-3098 **(e)** LCSET-3152 and **(f)** LCSET-3205.

Supplementary Figure - S6

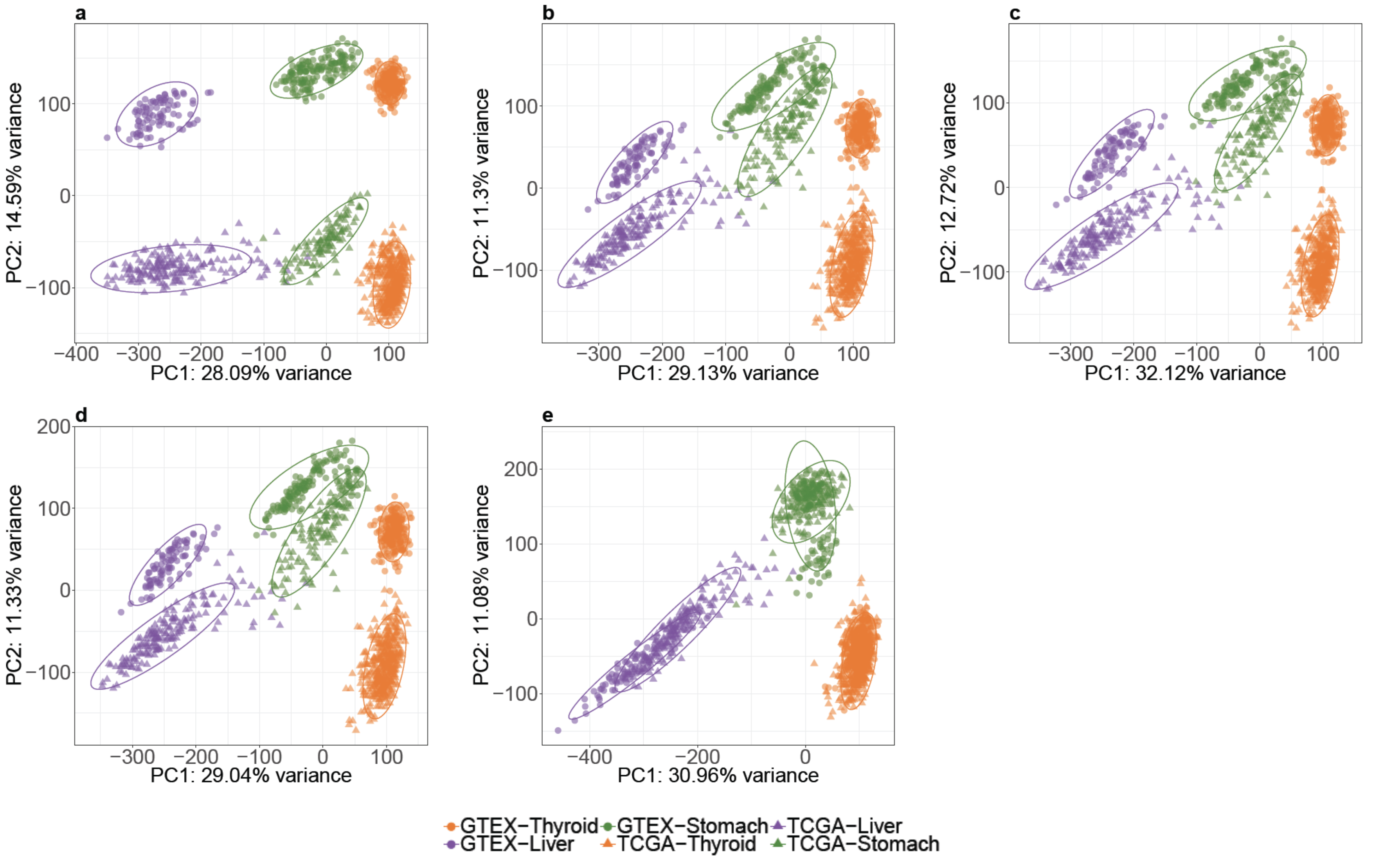

Supplementary Figure S6. Illustration of potential batch effects between TCGA and GTEx. Gene expression data for thyroid, liver and stomach were compared with gene expression data for their respective cancer types (THCA, LIHC and STAD) from TCGA. **(a)** GDC, **(b)** Xena/Toil, **(c)** Recount2, **(d)** normalized data from MSKCC (MSKCC) and **(e)** batch corrected data, after normalization from MSKCC (MSKCC Batch) showing reduced differences between TCGA and GTEx.

Supplementary Figure - S7

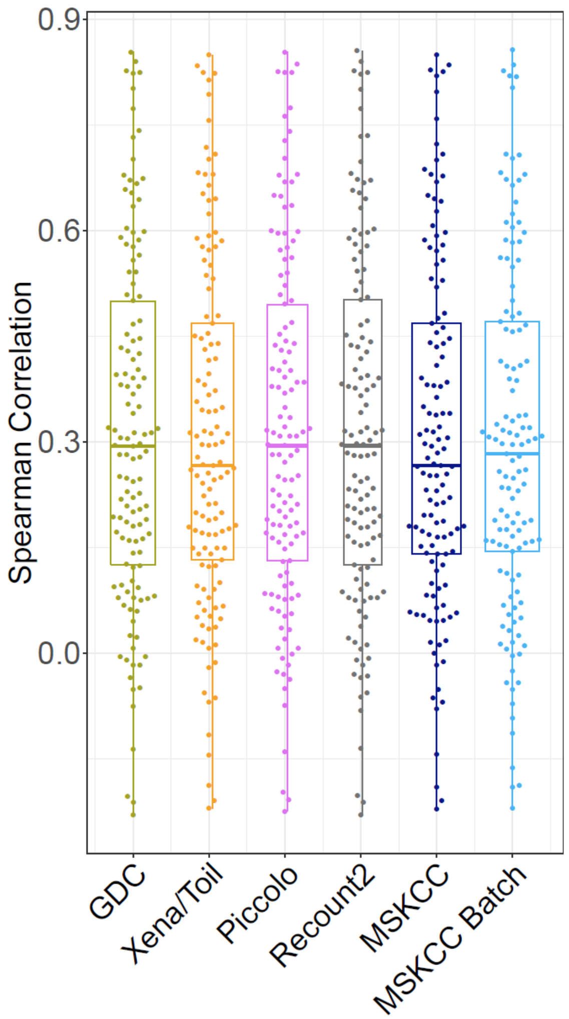

Supplementary Figure S7. Spearman correlation for gene expression data from TCGA and available protein abundance data.

Supplementary Figure - S8

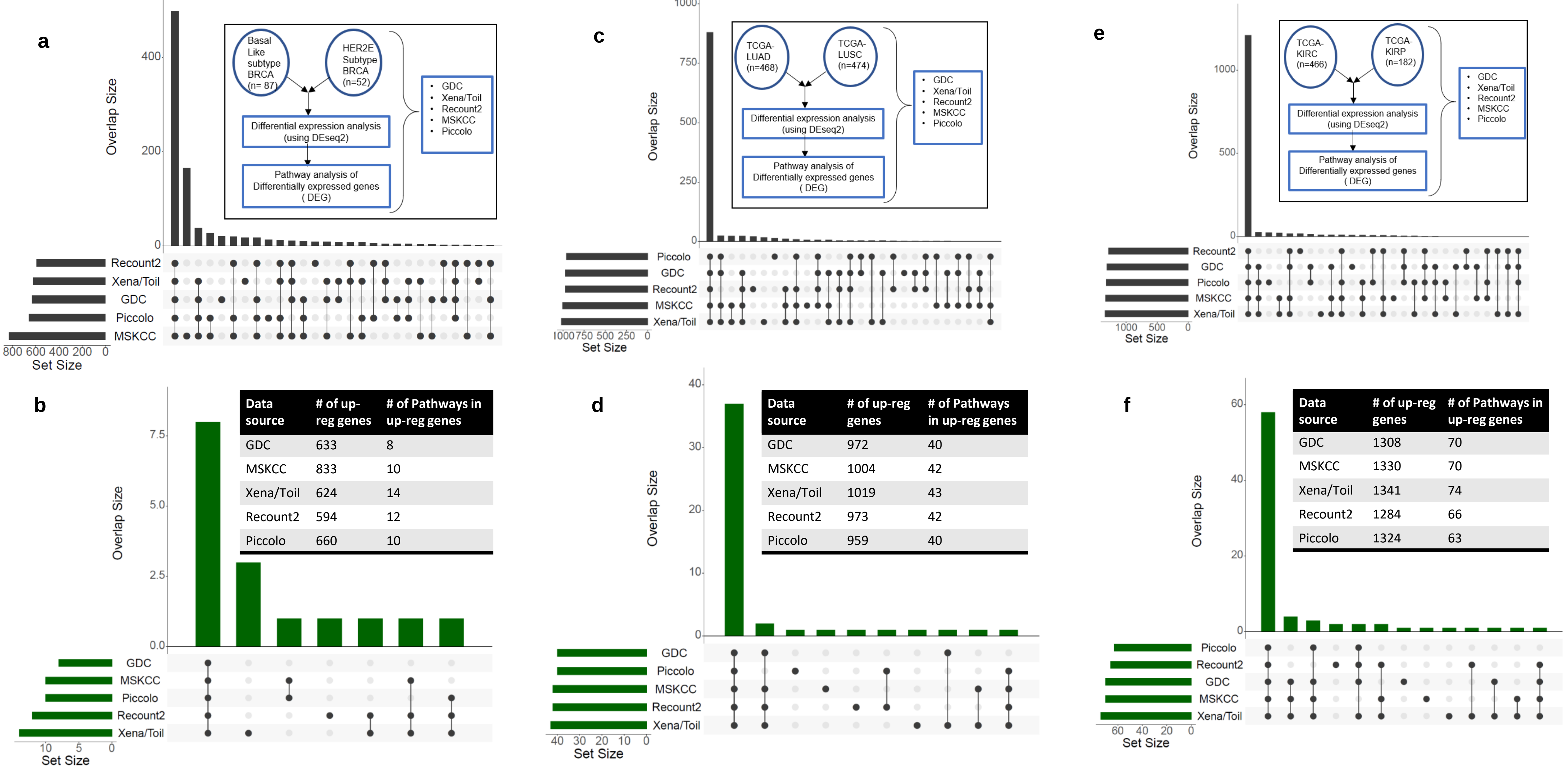

Data source	# of up-reg genes	# of Pathways in up-reg genes
GDC	633	8
MSKCC	833	10
Xena/Toil	624	14
Recount2	594	12
Piccolo	660	10

Data source	# of up-reg genes	# of Pathways in up-reg genes
GDC	972	40
MSKCC	1004	42
Xena/Toil	1019	43
Recount2	973	42
Piccolo	959	40

Data source	# of up-reg genes	# of Pathways in up-reg genes
GDC	1308	70
MSKCC	1330	70
Xena/Toil	1341	74
Recount2	1284	66
Piccolo	1324	63

Supplementary Figure S8. Upset plots showing differentially expressed genes and over-represented Reactome pathways in **(a, b)** Basal-like subtype compared to HER2-enriched(HER2E) subtype for TCGA-BRCA **(c, d)** Lung adenocarcinoma(TCGA-LUAD) compared to Lung squamous cell carcinoma (TCGA-LUSC) and **(e, f)** Kidney renal clear cell carcinoma (TCGA-KIRC) compared to Kidney renal papillary cell carcinoma(TCGA-KIRP) using same samples across all pipelines. Differentially expressed genes were obtained from DESeq2 (FDR < 0.05 & fold change >1).

Supplementary Figure - S9

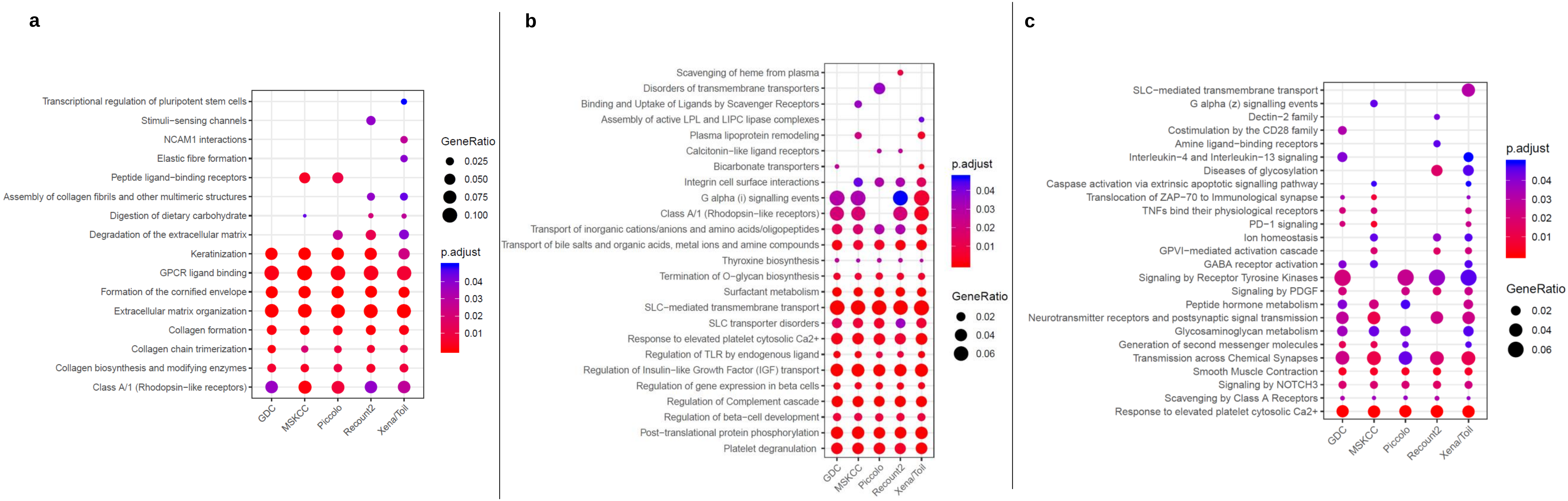

Supplementary Figure S9. Dot plots showing pathways over-represented in **(a)** Basal like subtype compared to HER2-enriched subtype of TCGA-BRCA samples , in **(b)** Lung adenocarcinoma(TCGA-LUAD) compared to Lung squamous cell carcinoma (TCGA-LUSC), and in (c) Kidney renal clear cell carcinoma (TCGA-KIRC) compared to Kidney renal papillary cell carcinoma (TCGA-KIRP) across all pipelines .(x-axis represents each pipeline, y-axis represents pathways, presence/absence of circle represents if the pathway was over-represented in respective pathway, size of circle represents geneRatio whereas color of circle represents the adjusted p-value, “GeneRatio” is the number of genes within the list which are annotated to the gene set divided by the size of list of genes of interest.)

Supplementary Figure - S10

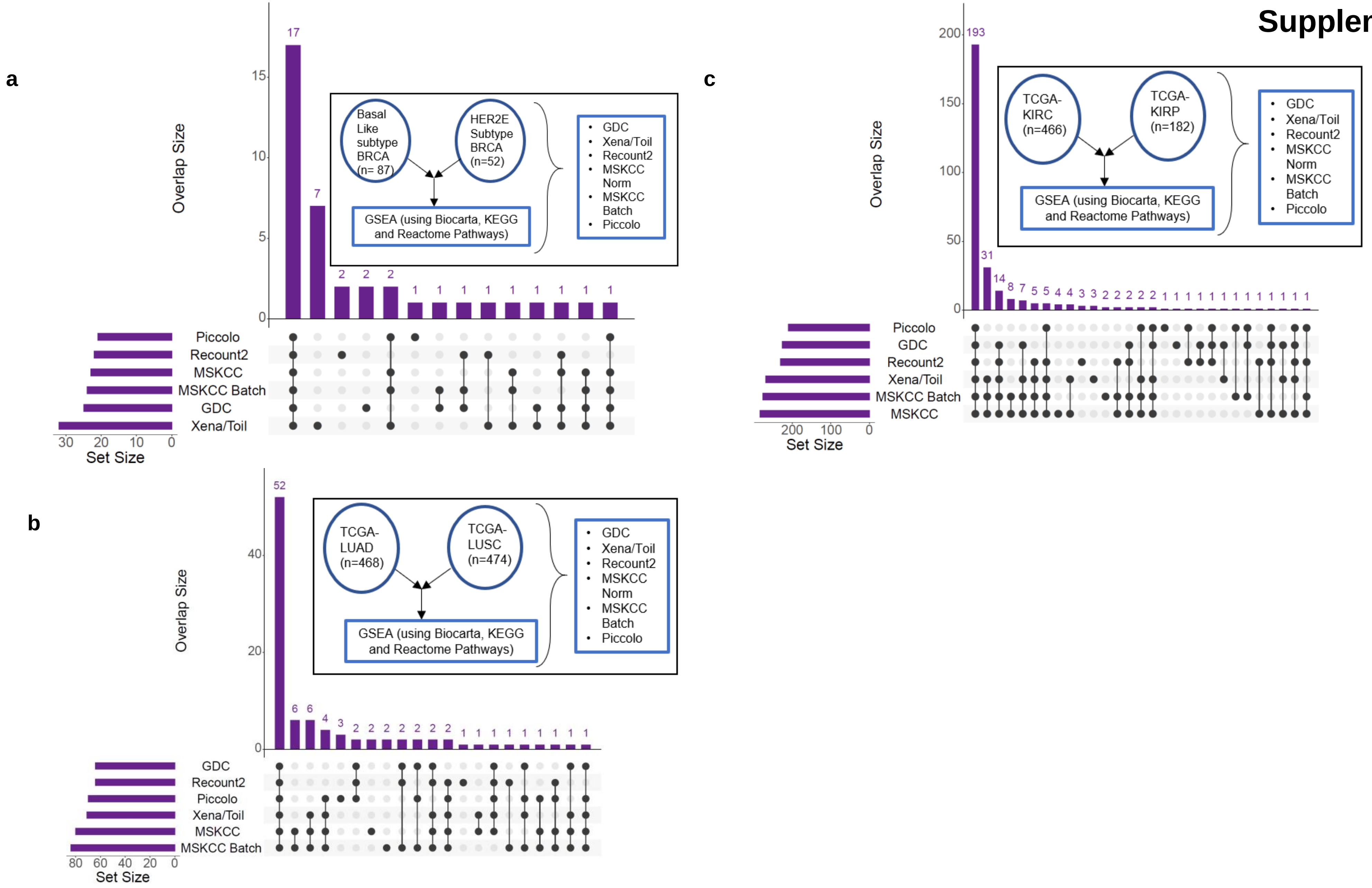

Supplementary Figure S10. Upset plots showing enriched pathways in **(a)**Basal-like subtype compared to HER2-enriched(HER2E) subtype for TCGA-BRCA **(b)** Lung adenocarcinoma(TCGA-LUAD) compared to Lung squamous cell carcinoma (TCGA-LUSC) and **(c)** Kidney renal clear cell carcinoma (TCGA-KIRC) compared to Kidney renal papillary cell carcinoma(TCGA-KIRP) obtained from GSEA analysis of log2(TPM) counts using same samples across all pipelines.

Supplementary Figure - S11

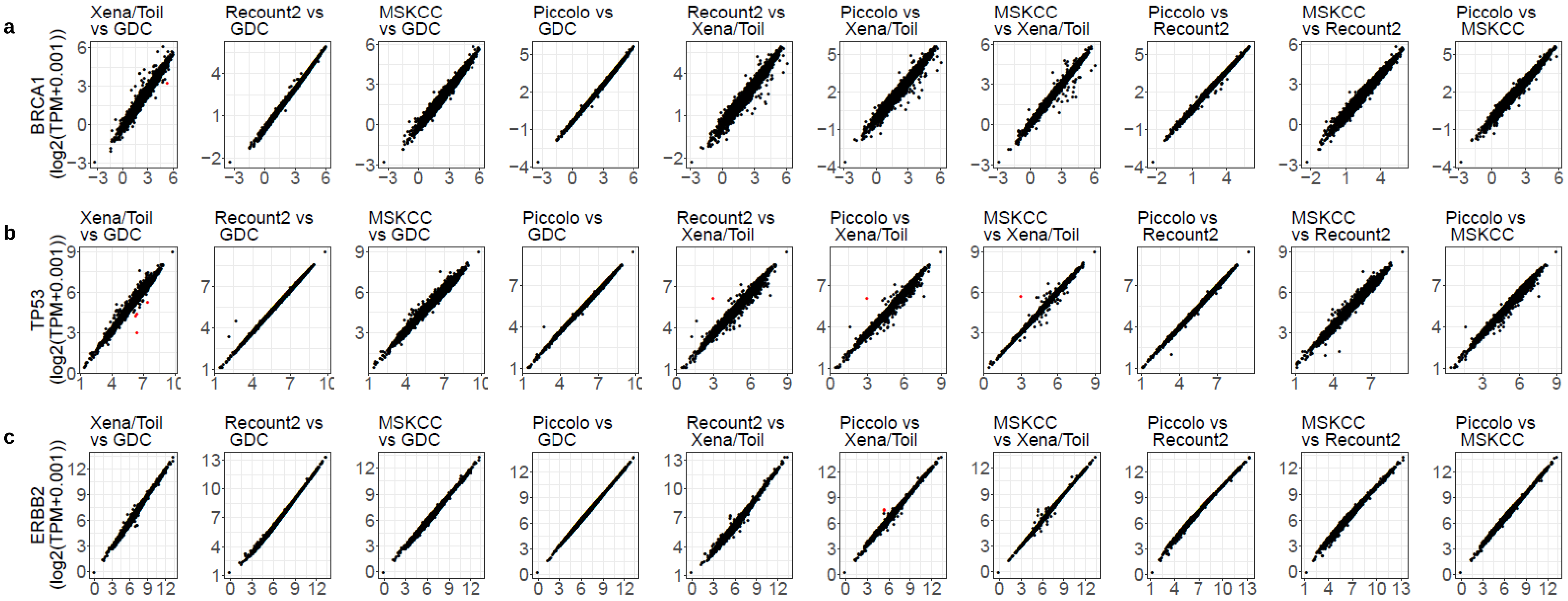

Supplementary Figure S11. **(a)** BRCA1, **(b)** TP53 and **(c)** ERBB2 (genes associates with Breast Cancer) show agreement among different pipelines. Panel titles specify the pipelines as Y-axis vs. X-axis. Red dots and black dots represent discordant and non-discordant samples respectively between two pipelines.

Supplementary Figure - S12

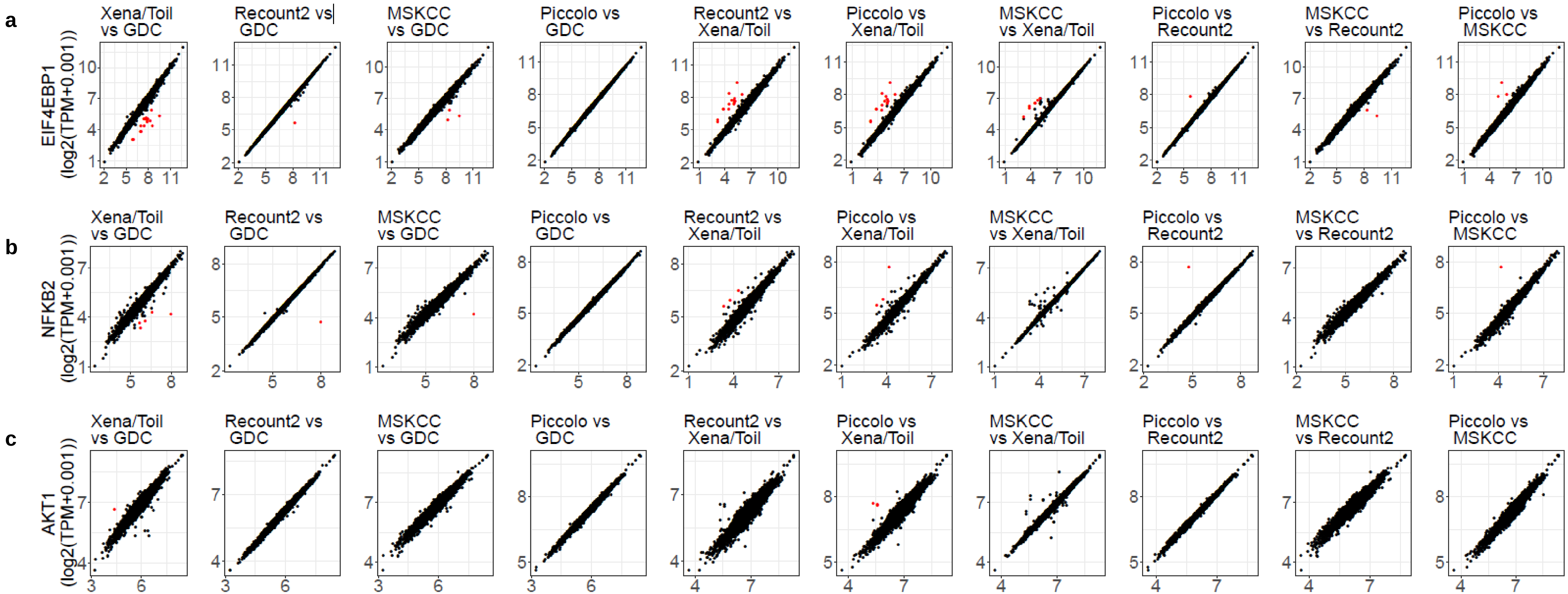

Supplementary Figure S12. **(a)** EIF4EBP1, **(b)** NFKB2 and **(c)** AKT1 (genes associated with prostate cancer) show agreement among different pipelines. Panel titles specify the pipelines as Y-axis vs. X-axis. Red dots and black dots represent discordant and non-discordant samples respectively between two pipelines.

Supplementary Figure - S13

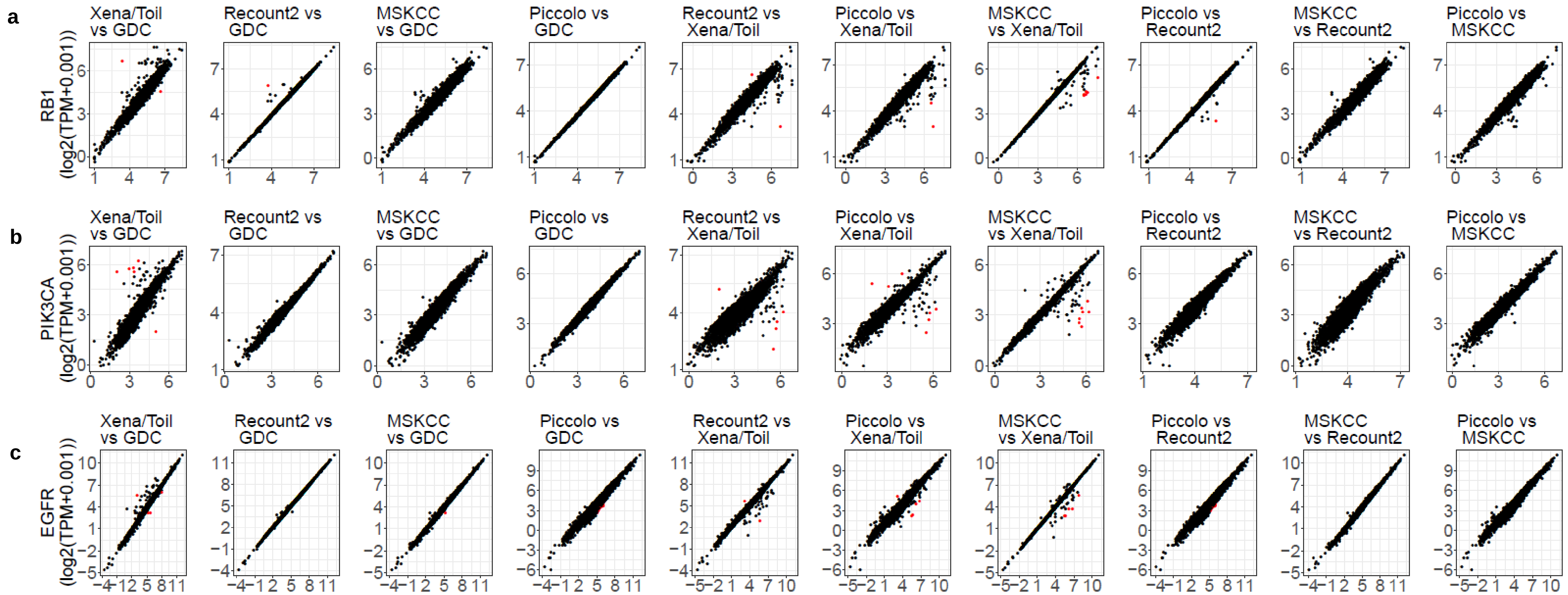

Supplementary Figure S13. **(a)** RB1, **(b)** PIK3CA and **(c)** EGFR (genes associated with glioblastoma) show agreement among different pipelines. Panel titles specify the pipelines as Y-axis vs. X-axis. Red dots and black dots represent discordant and non-discordant samples respectively between two pipelines.
